# Supplementary material for: Are carbon emissions trading and green financial instruments synergistic? -Comprehensive quantitative research based on content analysis
Source: PLoS One. 2024 Mar 7;19(3):e0298601. doi: 10.1371/journal.pone.0298601 (PMC10919601; doi:10.1371/journal.pone.0298601)
Supplement: S1 Data — (ZIP) [file pone.0298601.s001.zip › Data/地级市Z_F工作报告.zip!/╜¡╦╒/╤╬│╟╩╨/└┤╫╘╣1⁄2╓┌║┼í╛┬φ┐╦╩2╛▌═°í┐_2016.docx]

各位代表:

现在,我代表盐城市人民政府向大会作工作报告,请予审议,并请各位政协委员和其他列席人员提出意见。

“十二五”主要成就和2015年工作回顾

“十二五”时期,是我市综合实力大幅提升、城乡面貌显著改变、人民群众得益最多的五年。全市上下认真贯彻中央大政方针,坚决落实省委省政府和市委的决策部署,围绕“三先”追求、“四城”定位、“六大发展”战略布局,全面展开“建设新盐城、发展上台阶”的生动实践,顺利完成了“十二五”规划确定的主要目标任务,经济社会发展取得显著成就。

一、主动适应新常态,坚定不移推进稳增长调结构,经济总量质量大幅跃升。综合实力登上新台阶。与“十一五”末相比,地区生产总值从2333亿元增加到4213亿元,年均增长11.8%;人均地区生产总值9000美元,五年翻了一番;一般公共预算收入从191亿元增加到477.5亿元,年均增长20.1%。转型升级迈出新步伐。新能源及其装备、大数据、节能环保、新能源汽车等新兴产业主营业务收入年均增长30%以上。悦达集团成为千亿元级企业。建成大数据产业园、金融城、中汽中心盐城汽车试验场等一批现代服务业集聚区,服务业增加值年均增长13%。农业现代化进程综合得分苏北第一,联耕联种经营方式入选江苏农业创新十大举措。创新创业成为新引擎。建设市开发区、高新区、环保科技城、智慧科技城“两区两城”标志性产业创新高地,获批国家创新型试点城市。全社会研发投入占GDP比重比2010年提高一倍。实施“515”人才引进三年行动计划,全民创业蓬勃兴起,新增各类企业3.8万家。

二、抢抓国家战略机遇,坚定不移推进沿海开放开发,沿海发展取得重大进展。基础设施建设取得突破。盐城港“一港四区”建成万吨级码头泊位17个,货物吞吐量大幅增长。南洋机场改扩建工程顺利推进,临海高等级公路全线贯通,沿海综合交通体系基本形成。重大产业项目取得突破。列入省沿海开发五年推进计划的78个重点项目,累计完成投资3626亿元。50多家央企在盐投资85个重大项目,总投资1828亿元。沿海地区成为活力最强、增长最快的重要经济板块。沿海开放合作取得突破。“东向出海、南接沪深、西向开拓、北连央企”全方位开放格局初步构建。累计批准韩资项目689个,集聚台资企业878家。沪苏大丰产业联动集聚区及苏盐、盐常等合作共建园区加快建设,国家级滩涂综合开发试验区建设取得新的进展。

三、做大做强中心城市,坚定不移推进城乡统筹发展,城乡面貌发生巨大变化。中心城市现代化步伐加快。城市空间拓展,大丰撤市建区融入大市区。城市功能完善,五年实施城建重点项目416个,完成投入773亿元,城市道路等基础设施条件大为改观,一批城市综合体、特色街区、文化休闲场所建成使用。城市品质提升,五年新增公园绿地面积1520公顷,改造棚户区(城中村)260万平方米,建设各类保障性住房3.4万套。创成国家园林城市、国家卫生城市,省优秀管理城市创建通过考核验收。新型城镇化步伐加快。常住人口城镇化率由“十一五”末的52.5%提高到60.1%。推进城乡规划、产业发展、基础设施、就业保障、公共服务、社会治理“六个一体化”。五年新建改建干线公路1120公里、农村公路3800公里,社会公路总里程全省第一,村村开通客运班车。实施饮用水安全保障十大工程,城乡区域供水基本实现全覆盖。水利、电力、信息等基础设施建设得到加强。开展农村环境综合整治行动,完成1.5万个自然村庄整治任务,涌现一批“最美乡村”。

四、秉持绿色发展理念,坚定不移推进生态文明建设,环境品质得到极大提升。生产生活方式加快向绿色化转型。风力发电、光伏发电装机总量和增量均居全省第一,单位GDP能耗下降17%,完成省下达的节能减排任务。建制镇污水处理设施覆盖率达70%,农村生活垃圾集中收运系统基本建成使用。市区推广应用新能源车3400多辆,全面完成黄标车治理任务。环境综合治理成效明显。完成3万多条城乡河道整治任务。加强秸秆综合利用,秸秆禁烧连续两年实现“零火点”,空气质量持续保持全省最好、全国前列。绿色盐城建设有力推进。五年新增绿化造林面积90万亩,林木覆盖率达26%,生态效益指数全省第一。建成省级生态市,绿化模范城市创建通过国家考核验收,环保模范城市创建通过国家评估,我市成为国家可持续发展实验区。

五、坚持民生优先发展,坚定不移推进幸福盐城建设,群众生活质量持续改善。居民收入持续增长。城镇常住居民人均可支配收入28200元,农村常住居民人均可支配收入15748元,年均分别增长11.1%和13%。扶贫力度持续加大。322个经济薄弱村全部实现新“八有”目标,人均纯收入4000元以下的低收入人口全部脱贫。我市精准扶贫给经济社会发展带来巨大变化,得到了中央肯定。保障水平持续提高。城乡基本社会保险覆盖率97%。企业退休人员基本养老金、城乡居民基础养老金均增长75%。在全省率先实现省内异地就医全覆盖。住房公积金制度覆盖率65%。困难群众托底救助实现市域全覆盖。社会事业持续发展。全市新建、改扩建校舍450万平方米,义务教育发展基本均衡县(市、区)实现全覆盖。公立医院全面实行药品零差率销售,新型农村合作医疗、基本公共卫生服务、大病保险、基本药物制度实现全覆盖。建成4个全国文化先进县,打造了淮剧《小镇》等一批文艺精品。率先创成省公共体育服务示范区,建成健身步道248公里。厚德盐城成为城市新名片,有45人当选“中国好人”,数量全省第一。国防动员和国防后备力量建设、军民融合发展、人民防空、双拥共建等取得新的进展。社会治理持续加强。实现事故数量、信访总量、刑事案件“三个下降”,司法水平、公众安全感、法治满意度“三个上升”,连续四年获评全省社会管理综合治理先进市,被表彰为全国社会管理综合治理优秀市。

各位代表,2015年是实现“十二五”圆满收官的关键之年。这一年,我们积极应对经济下行压力加大、各种风险挑战增多的严峻形势,统筹推进稳增长、促改革、调结构、惠民生、防风险等各项工作,完成了市七届人大四次会议确定的各项目标任务。一是全力稳定经济运行。全市经济稳中有进、稳中向好、结构改善、质态提升。全年地区生产总值同比增长10.5%,一般公共预算收入增长14.2%,固定资产投资增长22.6%,社会消费品零售总额增长11.9%,规模以上工业增加值增长11.5%,城乡居民收入分别增长9.1%和9.3%。地区生产总值、规模以上工业增加值、固定资产投资、农村常住居民人均可支配收入等指标增幅位居全省前三位,全口径工业开票销售、服务业增加值、服务业投资、一般公共预算收入、进出口总额、城镇常住居民人均可支配收入、各项存贷款余额等指标增幅全省第一,一般公共预算收入跻身全国同类地级市前十强。二是大力调整产业结构。启动实施产业创新“十大工程”,计划总投资1700亿元,新兴产业主营业务收入增长30.7%。东台跻身全国工业百强县(市)。服务业增加值、投资、税收分别增长12.5%、25.5%、13.2%。电子商务交易额突破千亿元。中华麋鹿园创成5A级景区,黄海海滨森林公园创成国家级森林公园,九龙口国家级湿地公园创建通过专家评审。三是重点突破基础设施建设。启动实施城市提升“十大工程”,计划总投资1600亿元。市区55公里的内环高架快速路网工程全面开工建设,完成工程量的60%。跨区域引水的新水源地工程通过专家评审,并获得省政府批准,前期工作扎实推进。盐城至南京、徐州高速铁路(客运专线)当年立项、当年设计、当年开工建设,盐城至连云港快速铁路加快推进。阜建高速公路建成通车,实现“县县通高速”。启动建设南洋机场新航站楼。四是加快发展现代农业。新增高效设施农业21万亩,粮食生产实现“十二连增”。实施“接二连三”工程,创建国家级农产品加工示范基地2个、省级农产品加工集中区7个,农产品加工业产值突破2000亿元。

农村改革不断深化,80%以上的行政村开展土地承包经营权确权登记颁证,新发展家庭农场1062个。五是全面深化改革开放。大力推动行政管理体制、国有企业、财税金融、投融资体制和社会事业领域改革。市级行政权力事项、审批事项分别精简73.6%、55%,开展相对集中行政许可权试点工作。主动融入“一带一路”建设,扎实推进沿海开放开发,沿海板块对全市工业经济增长贡献率达94%。我市成为中韩两国政府确定的产业园地方合作重点城市,省政府出台专项政策支持中韩盐城产业园建设。盐城高新区获批国家级高新区,城南新区、建湖高新区创成省级高新区。六是着力保障改善民生。全市财政用于民生投入548亿元,新增财力的80%以上用于民生改善。20件民生实事工程完成投资113亿元。法治建设加强,社会和谐稳定。民生水平、民生发展、民生满意度指标和全面小康综合指标苏北第一。七是切实加强作风建设。深入开展“三严三实”专题教育,严格执行中央八项规定和省、市委十项规定,深化“四风”整治,公务接待、因公出国(境)、公车购置运行费用大幅下降,公车改革任务基本完成,群众对政府服务的满意度不断提升。坚持依法科学民主决策,自觉接受各方面监督,认真听取市人大代表、政协委员意见和建议,办理人大代表建议174件、政协委员提案228件。

各位代表,经过“十二五”时期的发展,我市走出一条增长与转型良性互动、发展与生态相得益彰、经济与社会协调并进的绿色发展有效路径,区域地位和城市影响力大幅提升,为“十三五”乃至今后更长时期发展奠定了坚实基础。成绩来之不易,这是省委省政府正确领导的结果,是在市委领导下全市人民开拓创新、团结奋斗的结果。在此,我谨代表市人民政府,向全市广大干部群众,向市人大代表、政协委员和离退休老同志,向各民主党派、工商联、人民团体和各界人士,向驻盐部队、驻盐单位,向所有关心和支持盐城发展的海内外朋友,致以崇高的敬意和衷心的感谢!

在肯定成绩的同时,我们也清醒地认识到面临的压力和挑战:发展不充分仍然是盐城最大的市情,经济稳健增长的基础还不牢固,重大产业项目不多,部分企业生产经营困难;结构性矛盾依然突出,传统产业比重较大,新产业经济支撑力不强,资源环境约束趋紧;制约发展的体制机制障碍仍然存在,发展活力还不够强;基本公共服务保障水平还不高,一些领域潜在风险不容忽视,机关作风和政府服务还有需要改进的地方。对存在的问题,我们一定高度重视,采取有力有效措施切实加以解决。

“十三五”时期主要目标任务

根据《中共盐城市委关于制定盐城市国民经济和社会发展第十三个五年规划的建议》,我们制定了“十三五”规划纲要(草案),提请本次大会审议。

“十三五”时期,是我市全面建成小康社会、推动“建设新盐城、发展上台阶”的关键时期。国家深入实施“一带一路”、长江经济带和江苏沿海发展等重大战略,省委省政府大力推进“一中心”“一基地”建设,为我市加快发展提供了难得机遇。我们必须抢抓战略机遇,坚定信心,克难奋进,努力实现经济社会更高质量、更有效率、更加公平、更可持续发展。

“十三五”时期,我们要全面贯彻党的十八大和十八届三中、四中、五中全会精神,深入贯彻习近平总书记系列重要讲话精神,按照“四个全面”战略布局,坚持发展第一要务,大力实施省“七大战略”“八项工程”,以提高发展质量和效益为中心,加快形成引领经济发展新常态的体制机制和发展方式,围绕“全面建成小康社会”总体目标,以“五大发展理念”为引领、“强富美高”为方向、“建设新盐城、发展上台阶”为定位、“绿色发展”为路径,秉持“三先”追求,统筹推进经济建设、政治建设、文化建设、社会建设、生态文明建设,加快建设创业、开放、生态、幸福、厚德的美丽盐城。

“十三五”时期我市经济社会发展的主要目标是:提前实现经济总量、城乡居民收入“两个翻番”,努力实现发展方式、产业结构“两个转变”,确保实现基本公共服务、托底救助保障“两个全覆盖”,全面建成小康社会。到2020年,综合实力显著增强,地区生产总值达7000亿元,年均增长10%左右;一般公共预算收入年均增长10%以上。转型升级成效明显,高新技术产业产值占规模以上工业产值比重达35%以上,服务业增加值占GDP比重达45.5%,现代农业发展水平全省领先。创新创业活力彰显,自主创新体系逐步建立,科技进步贡献率达61%,信息化发展水平总指数达到91。改革开放不断深化,重要领域和关键环节改革取得决定性成果,全方位开放新格局、开放型经济新体制基本形成。生态环境品质提升,能源资源使用效率大幅提高,主要污染物排放总量大幅减少,空气质量保持全省最好,建成国家生态市。民生幸福全面推进,城乡居民人均可支配收入分别达45000元、25000元,社会保障体系更加健全,社会治理水平和公众安全感明显提升。

实现“十三五”发展目标,必须全面贯彻创新、协调、绿色、开放、共享的发展理念,主动适应经济发展新常态,突出民生优先导向、改革创新导向、绿色发展导向,着力抓重点、攻难点、补短板,落实好五大重点任务。

建设万众创新的创业盐城。大力推动大众创业、万众创新,充分激发各类市场主体创新创业活力,新增创业主体28万人,努力拓展经济发展新空间。推进农业现代化,实现农村一二三产业融合发展。大力实施创新驱动发展战略,引进一批高水平研发中心,实现大中型工业企业研发机构全覆盖,基本形成以创新为引领支撑的经济体系和发展方式。大力推进产业创新“十大工程”,促进先进制造业与现代服务业深度融合发展,形成一批全国有竞争力和影响力的产业标杆,基本建成具有中高端水平的现代产业体系,产业结构实现从“二三一”到“三二一”的历史性跨越。

建设合作包容的开放盐城。打造全要素现代化综合交通,积极构建以高铁、空港、海港为标志的区域对外交通大通道,加快高速铁路建设,融入国家高速铁路网,缩短与上海、南京等中心城市的时空距离,真正迈入“高铁时代”。加快信息基础设施建设,全面进入“互联网时代”。优化沿海开发空间布局,统筹推进产业发展、港城和经济区建设,形成“一带”(临海高等级公路沿线黄金海岸经济带)、“两核”(大丰港城经济区和滨海港城经济区)、“三区”(东台滩涂经济区、亭湖射阳生态经济区和响水工业经济区)、“多节点”(沿海重点镇及有关开发园区)的开发格局。全力突破重大战略平台,加快中韩盐城产业园建设,建成第四代中外合作园区,成为两国创新创业合作新高地。推动园区、企业、城市、人才国际化,全面提升对外开放层次和水平。

建设绿色宜居的生态盐城。推行绿色生产方式,以绿色发展引领增长与转型,大力发展循环经济、低碳经济,开发利用绿色能源,壮大绿色产业,建设国家可持续发展实验区。开展绿色生活行动,推动全民在衣、食、住、行、游等方面形成绿色低碳、文明健康的生活方式。建设绿色美丽家园,加强丹顶鹤、麋鹿两个国家级自然保护区的保护与建设,申报世界自然遗产。强化生态资源保护,实施湿地生态修复工程,构建沿海综合防护林体系,打造绿色盐城靓丽品牌。

建设协调共享的幸福盐城。推动城市现代化和新型城镇化,实施城市提升“十大工程”,建成生态宜居、特色鲜明的现代化区域中心城市。构建“中心城市—县城—镇—新型农村社区”四级城乡体系,户籍人口城镇化率达56%。实施脱贫致富奔小康工程,到2019年,年纯收入低于6000元的低收入人口全部脱贫,经济薄弱村年集体收入全部达到18万元以上。推进公共服务共建共享,建成新水源地工程,从80公里外引进优质长江水源,彻底解决困扰我市多年的饮用水安全问题。对5600公里农村公路实施提档升级,实现农村公交“村村通”。加快推广应用清洁能源,天然气管网实现市区、镇区、中心社区全覆盖。全面提高教育、医疗卫生和社会保障水平,让全市人民过上更加美好的生活。

建设风清气正的厚德盐城。放大“盐城好人”效应,大力传承和弘扬铁军精神,提高市民文明素质,形成全社会崇德向善的好风气,人民群众的归属感、自豪感和幸福感明显增强。彰显盐阜文化特色,建成一批“非遗”保护示范基地,推出一批各具特色的文化旅游景区,打造一批全国知名的海盐文化品牌。创塑现代城市文明,加强公共文化、民主法治、社会信用体系建设,创新社会治理,建成全国文明城市,让城市精神成为推动盐城新一轮发展的强大力量。

2016年主要工作安排

2016年是“十三五”开局之年。做好今年各项工作,为“十三五”开好头、起好步至关重要。今年经济社会发展的主要预期目标是:地区生产总值增长10%左右,全社会研发投入占GDP比重达2%,一般公共预算收入增长11%,固定资产投资增长13%,社会消费品零售总额增长11%,外贸进出口总额增长10%,城乡居民收入增长10%左右,居民消费价格涨幅3%左右,城镇登记失业率控制在4%以内,完成省下达的资源环境约束性指标,人口自然增长率控制在4‰左右。

今年重点做好以下十项工作:

一、充分挖掘增长潜力,保持经济持续健康发展

发挥现有企业支撑作用。落实促进我市工业经济稳增长十条措施、金融支持实体经济发展六条意见,强化协调服务,推进会展拓市、外向拓市、电商拓市,充分释放有效产能,切实提高企业增长面、盈利面,确保规模以上工业增加值增长11%。支持东风悦达起亚汽车开拓市场、扩大销售。实施大企业(集团)培育、中小企业成长和百项新增长点培育工程,推进企业制造装备升级、互联网化提升工程,鼓励企业加强地产品配套,支持产业联盟开展配套协作。加强用地、用电、用工等要素保障,解决中小微企业融资难融资贵问题。

发挥投资消费拉动作用。优化投资结构,扩大产业、科技、重大基础设施投入,形成新的有效供给。深化项目载体突破年活动,加大重点项目推进力度,促进“双十”“三重”“八大类”重点工程项目早日开工建设、投产达效。实施技改升级工程,完成千万元以上技改项目500项,技改投资增长20%以上。积极培育消费新热点、新模式,扩大智能终端、智能服务等信息消费,壮大养老健康消费,促进旅游消费,推动文化消费升级。

发挥镇村经济基础作用。加快乡镇中小企业园建设,新建标准厂房200万平方米。大力培植“一镇一业”“一村一品”,重点建设40个特色产业基地、10个协作基地、30个特色农产品基地。突出“增收、减负、化债”,规范村级集体资金、资产、资源管理,切实增强经济薄弱村“造血”功能。

发挥全民创业促进作用。实施“创业盐城”行动计划,激发“八大主体”创新创业活力。推进登记注册制度便利化,设立“无障碍创业绿色通道”。扶持发展众创、众包、众扶、众筹等新业态、新模式。新发展私营企业2.2万家、个体工商户7.5万户。

二、更加突出创新驱动,促进产业结构优化升级

大力发展先进制造业。积极实施“中国制造2025”和“互联网+”盐城行动计划,坚持产业创新、重大项目、园区载体“三个聚焦”,打造产业链、创新链、价值链“三个链条”,强化要素、政策、服务“三个保障”,推进产业创新“十大工程”。做大做强新产业经济,积极培育电子信息、新能源、新能源汽车、节能环保、航空装备、海水淡化等产业,创建国家级大数据产业基地,加快发展互联网经济,新兴产业主营业务收入增长20%。开展智能制造三年行动计划,抓好50个智能制造重点项目、40个智能车间建设。推进建筑产业现代化,建筑业产值达到1660亿元。

加快发展现代服务业。大力发展科技金融、现代物流、电子商务、汽车后市场等专业化生产性服务业,培育壮大平台经济、工业设计等新兴业态,设立市级优秀工业设计奖,创建10个以上制造业服务化示范企业。积极发展现代商贸、文化休闲、健康养老、家庭服务等高品质生活性服务业,加快丹顶鹤生态旅游区、中华麋鹿园、大洋湾景区、大纵湖度假区、黄海国家森林公园等重点项目建设,开发一批旅游精品线路,全力突破沿海生态休闲旅游和乡村旅游。推进31个市级以上服务业集聚区发展,抓好100个亿元以上重点项目建设。

切实增强科技创新能力。实施创新型企业培育计划,推进企业“三自主”创新工程,新认定国家高新技术企业100家。推进产学研一体化,建设产业科技创新中心和先进制造业基地,打造3-5个产业创新示范园区,争创国家创新型城市、国家知识产权示范城市。深入实施“515”人才引进三年行动计划,引进一批高层次创新创业团队和专业人才,助力创新发展。

三、全力聚焦开放开发,推动沿海中部加快崛起

构建沿海现代综合交通体系。更大力度推进高速铁路建设,盐城至南通(通达上海)高速铁路年内开工建设,盐城至南京、徐州高速铁路开展路基桥梁施工,盐城至连云港快速铁路完成轨道铺设。加快大丰港区10万吨级深水航道和20万吨级散货泊位、滨海港区10万吨级码头泊位、射阳港区通用码头三期工程、响水港区5万吨级码头群建设,推动大丰、滨海港城经济区综合开发,支持大丰港区申报汽车整车进出口基地。继续实施南洋机场改扩建工程,加快建设新航站楼,加密飞往韩国、台湾等航线航班。建成盐丰高速、省道125市区段、滨海海港大道,推进省道233、351等干线公路建设。完善港城基础设施和功能配套,提升港产城联动发展水平。

提升开发园区建设水平。中韩盐城产业园建设总体规划争取国家批准,推动产业园政策集成创新和自由贸易区政策先行落地。加快“一园三区”基础设施建设,强化国际学校、国际医院、国际社区等功能配套,突破一批新兴产业重大项目。办好第15届泛黄海中日韩经济技术交流会。大力促进市开发区、高新区、环保科技城、智慧科技城等重点园区创新发展,支持有条件的开发区争创国家级开发区。建设综合保税区海关监管点、保税物流中心,推动沿海经济区设立海关特殊监管区和国家级检验检疫平台。推进沪苏大丰产业联动集聚区建设,提升与苏州、深圳、常州合作共建园区水平。加快临海高等级公路沿线产业带、城镇带、风光带建设,开发利用盐田建设用地资源,开工建设东台条子泥围垦二期工程。

加大招商引资力度。以百家央企、百家上市公司、百家跨国公司为重点,组织全天候、全方位产业招商活动。瞄准全球产业链、价值链的中高端,强化全产业链招商。突出各类开发园区主阵地作用,加强招商队伍建设,提高专业化招商水平,全力招引外资重大项目,加快建设中欧生态园、中德产业园,打造韩资密集区、台资新高地,全年利用外资力争突破10亿美元。

四、落实重大改革举措,激发经济社会发展活力

推进供给侧结构性改革。坚持用改革创新的办法,去产能、去库存、去杠杆、降成本、补短板,增加有效供给,提高全要素生产率。积极化解过剩产能,妥善处置严重资不抵债、长期停产半停产企业,加大企业不良贷款核销处置力度。深化住房制度、户籍制度改革,有序推进农民工市民化,释放住房需求,缩短房地产去化周期。积极争取开展投贷联动试点、不良资产证券化试点、区域金融改革创新试点。推进企业上市和发行企业债、公司债,提高直接融资比重。制定实施降低实体经济企业成本行动方案,切实把降低制度性交易成本、人工成本、企业税费负担、社会保险费、企业财务成本、电力价格、物流成本等政策措施落到实处。突出重大项目、基础设施、现代服务业、城乡居民收入、村级集体经济、城镇建设等重点,拉长补齐短板,实现经济社会全面协调发展。

推进行政管理体制改革。加大简政放权力度,完善“五张清单”。进一步清理投资项目审批前置事项,完成涉批中介机构脱钩改制,巩固和扩大“三证合一”“一照一码”等改革成果。坚持放管结合、优化服务,强化行政审批事中事后监管。实施不动产统一登记。

推进财税金融体制改革。加大政府性基金预算、国有资本经营预算与一般公共预算统筹力度。规范政府购买服务范围和购买目录,建立监督评估机制。完善市区财税体制,推进国、地税征管体制改革。加大招商引行力度,设立汽车金融公司,发展绿色金融。创新投融资方式,实施重点工程建设和融资计划管理,推广政府和社会资本合作(PPP)模式,开展林权、海域使用权等抵押融资试点工作。

推进国企国资改革。加快市属重点企业改革,加强资产整合,建立现代企业制度,完善国有资产监管体制。推动国资平台改革,做大做强城投、国投、交通控股等重点投融资平台,规范融资行为,建立风险预警机制,防范债务风险。制定发展混合所有制经济实施办法,鼓励和引导民间资本参与混合所有制改造。

五、转变农业发展方式,加快农业现代化进程

深入推进农业结构调整。优化种养结构,调粮、压棉、减油、扩特经。大力发展绿色农业、创汇农业,打造国省道沿线现代农业示范带、沿海百万亩现代渔业示范带,新增高效设施农业面积15万亩、设施渔业面积4万亩,建设20个省级出口农产品示范基地。大力发展休闲观光农业,带动更多农民增收致富。

促进农村一二三产业融合发展。深入实施“接二连三”工程,开展百家农业产业化龙头企业提升行动,重点培植销售超10亿元企业,农产品加工业产值突破2300亿元。开展“互联网+现代农业”行动,加快发展农村电子商务,推广“一村一品一店”。实施化肥农药减量化行动,健全农产品质量监管体系,打造10个区域竞争力强的农产品公共品牌。

提高农业物质装备和技术水平。开展耕地质量保护与提升行动,推进高标准农田和农村水利建设。积极争取列入国家“藏粮于地、藏粮于技”战略试点和耕地轮休轮作试点。推广农业新品种、新技术、新模式、新装备,加强粮食仓储能力建设,农业机械化水平达80%,农业科技进步贡献率达64%。

进一步深化农业农村改革。大力培育专业大户、合作组织、家庭农场等新型农业经营主体,新发展市级以上示范农民合作社300家、家庭农场600家。积极推进农业适度规模经营,联耕联种面积达到500万亩。开展农村土地及产权制度改革试点,完成农村土地承包经营权确权登记颁证工作。深化供销合作社综合改革,构建新型为农服务合作联社30家。

六、着力提升功能品质,推进城乡一体化发展

提升中心城市能级。坚持精明增长、集约高效、内涵发展,大力实施城市提升“十大工程”,完成年度投资380亿元。全面建成内环高架快速路网,开展沿线环境综合整治,提升城市形象。开工建设零换乘综合交通枢纽。改造提升世纪大道、解放路等主干道,实施东进路延伸等一批道路桥梁工程。开工建设跨区域引水的新水源地工程,完成盐龙湖水厂一期、通榆河取水口原水预处理净化等重点项目,保障居民饮用水安全。推进城南新区打造智慧、生态、人文、宜居新城,加快韩国城、万达广场等城市综合体建设,改造提升聚龙湖核心商圈,开工建设市旅游集散中心,进一步集聚新城区商气人气。改造棚户区(城中村)100万平方米、后街巷道50条以上,整治一批老旧小区,新建一批公共停车场,改善老城区人居环境。实施新洋港整治等城市防洪工程。加强信息化基础设施建设,推进海绵城市、地下综合管廊、静脉产业园建设,健全城市管理长效机制,完成城市管理综合执法改革任务。

提升新型城镇化质量。加快县(市)城建设,强化功能配套,促进产城融合。以国家新型城镇化综合试点为抓手,编制重点中心镇和新型农村社区总体规划、专项规划,加强30个重点镇、200个新型农村社区建设,实施一批污水处理、道路建设、供水管网、园林绿化等项目,集中打造东部沿海、中部沿204国道、西部沿边“三轴”城镇经济带。全市户籍人口城镇化率达49.6%。

提升城乡统筹发展水平。以试点镇村和示范带建设为抓手,持续推进“六个一体化”。推动城乡基础设施和公共服务设施互联互通、共建共享,加强镇村供水管网、城镇污水处理设施建设,实行公交一卡通,提高镇村公交通达率。启动实施5600公里农村公路提档升级三年行动计划,完成800公里农村公路和300座桥梁年度改造任务。改造农村危房30万平方米,完成农村改厕8万座。深入开展农村环境整治提升工程和“清洁乡村”行动,实现农村面貌更大改观。

七、加大生态建设力度,打造更加宜居宜业环境

推动低碳循环发展。实施绿色制造三年行动计划,开发利用绿色能源,推广清洁生产,实施30项节能技改工程,积极开展循环经济试点示范。推进低碳城市建设,倡导绿色出行,实施绿色建筑行动。推动绿色发展体制机制创新,建立节能量、碳排放权、排污权有偿使用交易机制。

持续改善环境质量。组织开展大气、水、土壤污染防治“三大战役”,着力解决损害群众健康和影响科学发展的突出环境问题。加强秸秆禁烧和综合利用,强化扬尘治理,全面淘汰小型燃煤锅炉。继续开展城乡河道综合整治三年行动计划,疏浚整治市区核心区河道19条、农村沟河1.5万条。

强化生态保护和修复。全面实施《盐城市绿化条例》,提升城市公园、街头游园绿化品质,推进沿海防护林、绿色村庄、生态景观廊道建设,全市新增成片造林面积5万亩。加强湿地自然保护区、湿地公园建设。积极申办省园艺博览会,加快国家级森林公园、湿地公园创建步伐。开展国家生态市创建工作,建成国家环保模范城市。

八、全面发展社会事业,提供更多优质公共服务

扎实推进文化建设。深入开展群众性精神文明创建活动,提高公民人文素质和社会文明程度。围绕打造串场河文化集聚区,开工建设杂技大世界、淮剧传习所、音乐厅等重点项目,建成水浒文化博物馆。实施文艺精品工程,扶持优秀文化产品创作。加强基层文化设施建设,完善市县镇村四级公共文化服务体系。大力发展文化产业,培育更多的文化市场主体和新型文化业态,文化产业增加值增长20%。

加强健康盐城建设。完善公立医院管理体制,加强医联体建设,构建分级诊疗体系。加强传染病、妇幼保健、精神病等专业医疗机构建设,推进市级卫生应急指挥中心、突发公共卫生事件处置中心建设。加强与知名医院合作,建立一批名医工作室和诊疗中心。完成42个镇卫生院和社区卫生服务中心、448个村卫生室和社区卫生服务站标准化建设。基本公共卫生经费由人均40元提高到50元。实施农村订单定向医学生免费培养工程,新增全科医生不少于350人。全面落实一对夫妇可生育两个孩子政策。突出体育惠民,加快体育休闲公园建设,新建健身步道200公里。

加快教育现代化建设。发展普惠性学前教育,实施新一轮学前教育五年行动计划和义务教育薄弱学校改造工程,新建、改扩建幼儿园86所、义务教育学校30所,财政用于教育的投入增长14%以上。进一步整合职业教育资源,推进产教融合。成立盐城幼儿师范高等专科学校,提升高等教育水平。加强与外地名校合作办学,提高教育教学质量。

关心支持老干部、关工委工作,更加关爱留守老人、留守妇女、留守儿童,发展慈善、残疾人事业,加强防震减灾、气象、援疆、援青等工作。

积极支持深化国防和军队改革,深入开展全民国防教育,加强国防动员、国防后备力量和人民防空建设,推进军民融合深度发展,广泛开展双拥共建活动,巩固和发展军政军民团结。

九、持续加强民生建设,提升群众幸福感获得感

促进居民收入持续增长。深化收入分配制度改革,鼓励创业扩大经营性收入,托底救助提高转移性收入,推进企业普遍建立工资集体协商制度,适时提高最低工资标准和低收入人群补贴标准。着力抓好就业增收,做好农民、中低收入者和困难家庭等重点群体就业工作,城镇新增就业9万人,援助4000名就业困难人员再就业。

更大力度推进新一轮扶贫开发。全面启动实施脱贫致富奔小康工程,聚焦低收入群体、经济薄弱村和重点片区,做好建档立卡等基础工作,加大投入,下足力道,实施精准扶贫、精准脱贫,确保完成年度脱贫任务。

提高社会保障水平。实施全民参保登记计划,巩固机关事业单位养老保险制度改革成果。深化医保付费方式改革,健全城乡居民大病保险制度,城乡居民医保财政补助标准由人均380元提高到425元。逐步推进各项保险市区统筹,加快社会保障卡发放和运用,加强社保服务平台建设。开展住房公积金扩面行动。落实低保提标、医疗救助政策,完善临时救助制度,实现“救急难”运行机制市域全覆盖。建设大市区居家养老智能化服务中心,鼓励社会力量兴办养老机构,推进医养融合发展。

加强和创新社会治理。坚持社会治理法治化、精细化,组织实施“七五”普法规划,加强法律服务和法律援助,强化基层基础,推进“阳光信访”,完善社会矛盾纠纷多元化解机制。加快智慧平安城市建设,创新升级立体化现代化社会治安防控体系,提高突发事件应急处置能力。健全食品药品安全监管机制,切实加大专项整治和综合治理力度。

扎实办好民生实事。突出饮水安全、收入增长、交通出行、防洪保安、托底保障、教育卫生、文化体育、棚户区改造、环境整治等方面,为民兴办20件实事,完成年度投资181亿元,增长60%,集中解决一批群众最关心、最直接、最现实的利益问题。我们将以高度负责的精神,全力聚焦民生实事项目,加大财政投入,严格落实责任,强化督查考核,确保全面完成。

十、坚决守住“四条底线”,落实“四个最严格”制度

严守“四条底线”。坚决守住生产安全和公共安全底线,严格落实安全生产责任制和责任追究制,推进企业安全生产标准化建设,深入开展重点园区和危化品、交通运输、建筑施工等重点行业专项整治,构建全过程、无缝隙公共安全监管体系。坚决守住生态环保底线,决不以牺牲环境为代价换取一时的经济增长,决不把降低环保门槛作为招商引资的优惠条件。实施最严格的生态空间管制,严把产业导向关、项目准入关、节能减排关。坚决守住耕地保护和节约用地底线,实现建设用地总量和土地开发强度“双控”、节地水平和产出效益“双提升”目标。坚决守住民生保障和社会稳定底线,编实筑牢社会保障体系“网眼”,兜住底线,保住基本民生。深入开展社会稳定隐患排查活动,切实防范非法集资、征收拆迁、工程款兑付、企业兼并重组等潜在风险,全力维护社会和谐稳定。

强化刚性约束。实行最严格的环境保护制度,全面清理整治环境保护违法违规建设项目,完成省下达的主要污染物排放量削减指标。实行最严格的水资源管理制度,加强水资源开发利用控制红线管理,积极创建国家节水型城市,万元GDP用水量下降5%,万元工业增加值用水量下降4%。实行最严格的节约用地制度,加强土地利用总体规划实施管理,全面清理处置闲置低效土地,提高建设用地利用效率。建设用地地均GDP增长10%。实行最严格的耕地保护制度,强化土地用途管制,严格控制非农业建设占用耕地,确保耕地保有量1220万亩以上,基本农田面积1145万亩以上。

切实加强政府自身建设

面对新形势、新任务和人民群众新期待,必须以改革创新精神加强政府自身建设,提高政府执行力和公信力,努力开创政府工作新局面。

一、强化依法行政。牢固树立法治意识,依法行使权力、履行职责,坚决查纠行政不作为、乱作为、慢作为。坚持依法科学民主决策,完善行政决策程序和决策咨询机制,开展重大政策举措第三方评估试点。推进综合执法,全面落实行政执法责任制。自觉接受人大法律监督、工作监督和政协民主监督,重视社会公众监督、新闻媒体监督,让行政权力在阳光下运行。认真办理人大代表建议、政协委员提案,切实改进政府工作。

二、优化发展环境。加强服务型政府建设,进一步转变职能,减少行政审批事项,提高投资贸易便利化水平,营造便捷高效的政务环境。保护企业产权和投资者合法权益,提振企业投资信心,营造公平正义的法治环境。规范中介服务和涉企收费行为,强化市场监管,营造诚信公平的市场环境。大力倡导创新创业、亲商安商,营造开放包容的人文环境。

三、深化作风建设。严格执行中央八项规定和省、市委十项规定,健全长效机制,巩固扩大作风建设成果。强化宗旨意识,更好地为基层、企业和群众服务,帮助解决实际问题。践行“三严三实”,强化责任担当,对确定的目标任务抓铁有痕、善做善成。创新政务服务方式,推进政务云平台建设,开展万人评议机关活动,强化督查考核、效能监察、行政问责,提高人民群众对政府工作的满意度。

四、建设清廉政府。强化领导干部“一岗双责”,推动政府系统党风廉政建设责任制落到实处。加强政府内部权力制约,坚持用制度管权、管事、管人,加快形成不敢腐、不能腐、不想腐的体制机制。加强审计监督,对公共资金、国有资产、国有资源和领导干部履行经济责任情况实行审计全覆盖。严查重点领域、关键环节违法违纪案件,严查发生在群众身边的“四风”和腐败问题。加强党风廉政教育,严格执行《准则》、《条例》,推动各级干部始终保持清正廉洁的良好形象。

各位代表!实现“十三五”及今年发展目标,任务艰巨,责任重大,使命光荣。让我们紧密团结在以习近平同志为核心的党中央周围,在省委省政府和市委的坚强领导下,紧紧依靠全市人民,振奋精神、凝心聚力,开拓进取、奋发有为,全力推动经济社会发展迈上新台阶,为建设创业、开放、生态、幸福、厚德的美丽盐城而努力!
